# Oral microbiome associated with differential ratios of *Porphyromonas gingivalis* and *Streptococcus cristatus*

**DOI:** 10.1128/spectrum.03482-23

**Published:** 2024-01-17

**Authors:** Qingguo Wang, Bing-Yan Wang, Siddharth Pratap, Hua Xie

**Affiliations:** 1 School of Applied Computational Sciences, Meharry Medical College, Nashville, Tennessee, USA; 2 School of Dentistry, University of Texas Health Science Center at Houston, Houston, Texas, USA; 3 School of Medicine, Meharry Medical College, Nashville, Tennessee, USA; 4 School of Dentistry, Meharry Medical College, Nashville, Tennessee, USA; The Ohio State University College of Dentistry, Columbus, Ohio, USA

**Keywords:** *Porphyromonas gingivalis*, periodontitis, oral microbiome, shotgun metagenomic sequencing

## Abstract

Periodontitis has recently been defined as a dysbiotic disease caused by an imbalanced oral microbiota. The transition from commensal microbial communities to periodontitis-associated ones requires colonization by specific pathogens, including *Porphyromonas gingivalis*. We previously reported an antagonistic relationship between *Streptococcus cristatus* and *P. gingivalis*. To determine the role of *S. cristatus* in altering the interactions of *P. gingivalis* with other oral bacteria in a complex context, we collected dental plaque samples from patients with periodontitis and assigned them to two groups based on the ratios of *S. cristatus* and *P. gingivalis*. We then characterized the microbial profiles of the dental plaque samples using shotgun metagenomic sequencing and compared the oral microbial composition and functional capabilities of the group with high *S. cristatus-P. gingivalis* ratios with the low ratio group. Taxonomic annotation revealed significant differences in the microbial composition at both the genus and species levels between the low and high *S. cristatus-P. gingivalis* ratio groups. Notably, a higher microbial diversity was observed in the samples with low *S. cristatus-P. gingivalis* ratios. Furthermore, the antibiotic resistance gene profiles of the two groups were also distinct, with a significantly increased abundance of the genes in the dental plaque samples with low *S. cristatus-P. gingivalis* ratios. It, therefore, indicates that the *S. cristatus-P. gingivalis* ratios influenced the virulence potential of the oral microbiome. Our work shows that enhancing the *S. cristatus-P. gingivalis* ratio in oral microbial communities can be an attractive approach for revising the dysbiotic oral microbiome.

**IMPORTANCE:**

Periodontitis, one of the most common chronic diseases, is linked to several systemic diseases, such as cardiovascular disease and diabetes. Although *Porphyromonas gingivalis* is a keystone pathogen that causes periodontitis, its levels, interactions with accessory bacteria and pathobionts in the oral microbiome, and its association with the pathogenic potential of the microbial communities are still not well understood. In this study, we revealed the role of *Streptococcus cristatus* and the ratios of *S. cristatus* and *P. gingivalis* in modulating the oral microbiome to facilitate a deeper understanding of periodontitis and its progression. The study has important clinical implications as it laid a foundation for developing novel non-antibiotic therapies against *P. gingivalis* and improving the efficiency of periodontal treatments.

## INTRODUCTION

Periodontitis is one of the most common diseases in humans, affecting approximately 42% of adults aged 30 years and older in the USA. The prevalence of periodontitis is influenced by multiple factors including age, gender, socioeconomic status, and race/ethnicity (1). The oral microbiota, which is composed of hundreds of bacterial species and phylotypes, is considered an important contributor to the development of periodontitis (2, 3). Thus, understanding the effects of individual bacterial species and their interrelationship in complex oral microbial communities is crucial in identifying microbiological factors associated with susceptibility to and severity of periodontitis.

*Porphyromonas gingivalis* is known to play a vital role in the development of dysbiotic microbial communities and can disrupt host-microbial homeostasis and induce inflammatory responses by interacting with other oral bacteria. Therefore, it is considered a keystone pathogen of periodontitis (4, 5). We previously reported an antagonistic relationship between *Streptococcus cristatus* and *P. gingivalis* and identified *S. cristatus* ArcA and a streptococcal ArcA-derived anti-*P*. *gingivalis* peptide (SAPP) that can effectively inhibit biofilm formation, invasion, and gingipain enzymatic activity of *P. gingivalis* (6–10). We also demonstrated that SAPP could reduce *P. gingivalis*-induced alveolar bone loss in a mouse model (11, 12) and eliminate *P. gingivalis* and other periodontitis-associated bacteria from oral microbial communities using *ex vivo* assays (11, 13). Moreover, our clinical studies showed a negative correlation between the distributions of *S. cristatus* and *P. gingivalis* in dental plaques derived from patients with periodontitis and higher *S. cristatus-P. gingivalis* ratios in dental plaque samples collected from Caucasian American (CA) patients with periodontitis, compared with those from African American (AA) and Hispanic American (HA) patients (14, 15). However, it is unclear whether the *S. cristatus-P. gingivalis* ratios are associated with particular oral microbial profiles and functions and whether the *S. cristatus-P. gingivalis* ratio can be used as a clinical parameter to predict the progression of periodontitis.

The goal of this work was to identify the similarities and differences shared among dental samples with relatively high or low *S. cristatus-P. gingivalis* ratios. Our overall hypothesis is that the ratio of *S. cristatus-P. gingivalis* may play a vital role in the pathogenic properties of the oral microbiome and contribute to the pathogenicity of the oral microbiome. Previously, the abundances of *P. gingivalis*, *S. cristatus*, and total bacteria in dental plaque samples from patients with periodontitis were determined using quantitative polymerase chain reaction (qPCR), and the *S. cristatus-P. gingivalis* ratio for each sample was calculated (14). In this study, we profiled microbial communities at the species level and characterized the functional and biological process of microbial communities using metagenome shotgun sequencing. By comparing the abundance and diversity of microbial taxa in dental plaque samples with relatively high *S. cristatus-P. gingivalis* ratios to those with low ratios, we observed differential composition and abundance of oral microbial communities with different *S. cristatus-P. gingivalis* ratios. Additionally, we found an increased abundance of antibiotic-resistance genes in the samples with low *S. cristatus-P. gingivalis* ratios. Collectively, the comparative study revealed an involvement of *S. cristatus* and *P. gingivalis* ratios in regulating the pathogenicity of oral microbial communities.

## MATERIALS AND METHODS

### Study cohorts

Participants were screened during routine dental visits in a clinic in the School of Dentistry, University of Texas Health Science Center, Houston between 2017 and 2022. Individuals aged 21–75 years were enrolled after the initial periodontal examination, which included the determination of the plaque index (PI) and bleeding on probing (BOP) (16). Radiographs were obtained during the screening process to assess bone loss. Clinical oral examinations were performed by trained dental examiners who were faculty members at the School of Dentistry. The examiners are calibrated in the diagnosis of periodontitis annually. All study participants were diagnosed with stage II or III generalized periodontitis, regardless of their grade, based on the 2017 World Workshop Classification (17, 18). The enrolled patients also met the following criteria: ≤4 teeth loss due to periodontitis, interdental clinical attachment level (CAL) ≥ 3 mm, pocket depth (PD) ≥ 5 mm at two or more teeth in different quadrants, and radiographic bone loss ≥ 15%. Other criteria for study participation were as follows: (i) no scaling and root planing within the previous year or periodontal surgeries in the previous 5 years; (ii) no antibiotic therapy in the previous 6 months; and (iii) no pregnancy. Information on demographics and self-reported dental and medical histories of the participants were obtained from the electronic health records.

### Dental plaque sample collection

Dental plaque samples, including supra and subgingival dental plaques, were collected by board-certified periodontists using sterile paper points prior to any dental treatment and labeled numerically according to the sampling sequences. The paper points were placed in ≥5 mm pockets in different quadrants for 1 min and then immersed immediately in an Eppendorf tube with 0.5 mL of Tris-EDTA (TE) buffer (pH 7.5) (15). Bacterial pellets were harvested by centrifugation and then resuspended in 100 µL TE buffer.

### Sequencing and quality control

Samples were sent to Novogene Co. (Sacramento, CA, USA) for metagenomic sequencing. Briefly, DNA extracted from human dental plaques was randomly sheared into short fragments, and the resulting fragments were end-repaired, A-tailed, and ligated using Illumina adapters. The fragments with adapters were subsequently amplified using PCR, followed by size selection and purification. Quality control of the library was conducted using Qubit (≥20 ng and ≥10 ng/µL), and quantification and size distribution detection were performed using real-time PCR and a bioanalyzer, respectively. The quantified libraries were pooled and sequenced using an Illumina NovaSeq high throughput sequencer by Novogene Corporation, Inc., with a paired-end sequencing length of 150 bp and an output of ~6 GB of raw data per sample.

### Data preprocessing

The average size of raw data generated per sample was 6.276 GB. To ensure the accuracy and reliability of the subsequent data analysis, all low-quality bases (*Q*-value ≤ 38) that exceed a certain threshold (40 bp), as well as reads containing N nucleotides over 10 bp and those overlapping with adapters > 15 bp were trimmed. To minimize host DNA contamination, raw reads that were mapped to the human reference genome were discarded using the Bowtie2 software (19). After quality control and host filtering, the size of clean data was 6.275 GB per sample, with 96.66% and 92.37% of bases having quality scores greater than 20 and 30, respectively.

### Gene prediction and abundance analysis

The MetaGeneMark software was used to predict open reading frames (ORFs) from scaftigs (≥500 bp) (20). ORFs less than 100 nt were discarded. To generate gene catalogs, the remaining ORFs were dereplicated using CD-HIT (21, 22) in default settings (i.e., identity = 95% and coverage = 90%). To calculate the gene quantity, the clean data were mapped to the gene catalog using Bowtie2 (parameters: –end-to-end, –sensitive, –I 200, –X 400). Gene abundance (*G_k_*) was calculated using the following formula:


$$G_{k}=\frac{r_{k}}{L_{k}}\frac{1}{\sum_{i=1}^{n}\frac{r_{i}}{L_{i}}}$$

where *r* represents the number of mapped reads and *L* represents gene length. Downstream analyses were performed based on the abundance of the genes in the catalogs.

### Taxonomy annotation

The software tool DIAMOND (version 0.9.9.110) (23) was used to align the sequences of the identified genes to those of bacteria, fungi, archaea, and viruses extracted from NCBI’s NR database (version 2018-01-02). MEGAN software was used to taxonomically annotate each metagenomic homolog (24). The sum of the abundance of genes annotated as a species in a sample was used as the abundance estimate of that species in that sample. Based on the abundance of each taxonomic level, various analyses were performed, including heatmap of abundance, principal coordinate analysis (PCoA), principal component analysis (PCA), and non-metric multidimensional scaling (NMDS) analysis, which is an indirect gradient analysis approach that produces ordination based on a distance matrix. R package ade4 (version 3.2.1) was used to perform PCA analysis and R package vegan (version 2.15.3) was used for PCoA and NMDS analyses.

### Functional analysis

The identified gene sequences were aligned with those in functional databases utilizing the DIAMOND software (version 0.9.9.110) (23), with parameter settings: blastp, -e 1e-5. The functional databases used in this study include Comprehensive Antibiotic Resistance Database (CARD) (25), Carbohydrate-Active Enzymes Database (CAZy) (26), Kyoto Encyclopedia of Genes and Genomes (KEGG) (27), and Genealogy of Genes: Non-supervised Orthologous Groups (eggNOG) (28). Based on the sequence alignment results, the best Blast hits were selected for subsequent analysis, and the relative abundance were calculated at different functional levels.

### Statistical analysis

Statistical analyses were performed using the package scipy.stats in SciPy (version 1.4.1), an open-source Python library for scientific computing. Independent two-sample *t*-tests were performed to compare samples with high *S. cristatus-P. gingivalis* ratios to those with low ratios. The *t*-tests were two-sided, and an assumption of identical variances on the sample distributions was used (https://github.com/qwangmsk/Oral-Metagenomics). A *P*-value of <0.05 was considered statistically significant.

## RESULTS

### Characteristics of the study cohort

We previously investigated the abundances of several well-studied oral bacteria, including keystone pathogens, accessory pathogens, and pathobionts, in dental plaques derived from patients with periodontitis using qPCR (29, 30). The qPCR results demonstrated that the ratio of *S. cristatus* to *P. gingivalis* is significantly higher in Caucasian Americans than in Hispanic Americans and African Americans, suggesting that higher levels of *P. gingivalis* and lower ratios of *S. cristatus* to *P. gingivalis* may contribute to periodontal health disparities (14). To examine the role of *P. gingivalis* and its antagonistic species *S. cristatus* in the oral microbiota composition, we selected 14 samples with relatively low *S. cristatus-P. gingivalis* ratios (<1) and 16 samples with higher ratios (>100) based on the results of qPCR for shotgun metagenomic sequencing. The general characteristics of the participants are shown in Table 1. No significant differences in gender, age, BOP, PI, and the number of teeth between the two groups were observed (Table 1).

**TABLE 1:** Characteristics of the study cohort

	*S. cristatus-P. gingivalis* ratio		
Characteristics	<1	>100	*P*-value
Gender (male/female)	6/8	12/4	0.078
Age (year, mean ± SD)	54.6 ± 13.3	64.1 ± 12.2	0.052
BOP (%, mean ± SD)^*a*^	52.9 ± 22.3	44.6 ± 31.0	0.413
PI (%, mean ± SD)^*b*^	69.1 ± 27.2	69.7 ± 31.5	0.958
Teeth number (mean ± SD) *^c^*	26.7 ± 1.9	25.9 ± 1.8	0.232

^*a*^ BOP, bleeding on probing.

^*b*^ PI, modified O’Leary plaque index.

^*c*^ Teeth number is based on a total of 32.

### Diversity and similarity of the oral microbiota with different *S. cristatus-P. gingivalis* ratios

**Fig 1:**
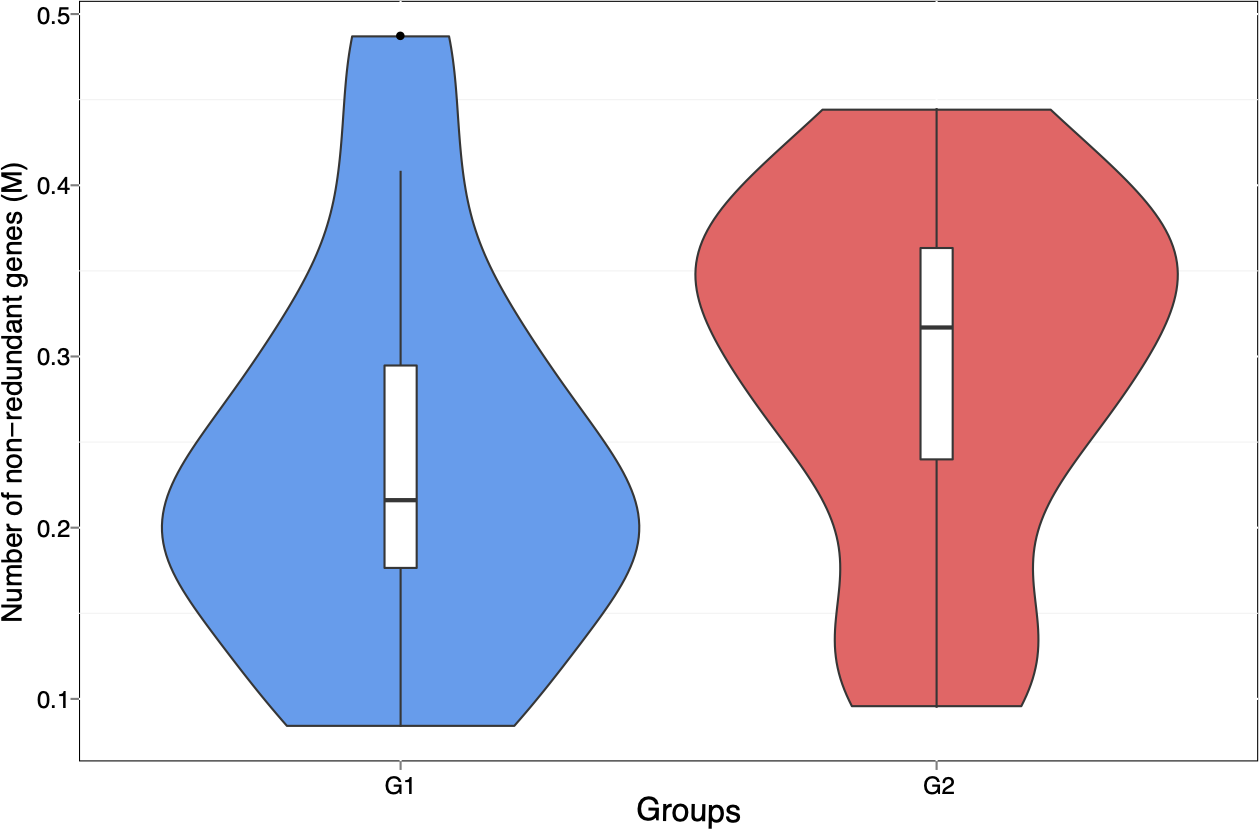
Comparison of the non-redundant genes between samples with different *S. cristatus-P. gingivalis* ratios. Gene abundance was calculated based on the total number of mapped reads and gene lengths. The blue violin represents the richness and abundance of non-redundant genes in the samples with low *S. cristatus-P. gingivalis* ratios (G1). The red violin represents the richness and abundance of non-redundant genes in the samples with high *S. cristatus-P. gingivalis* ratios (G2).

**Fig 2:**
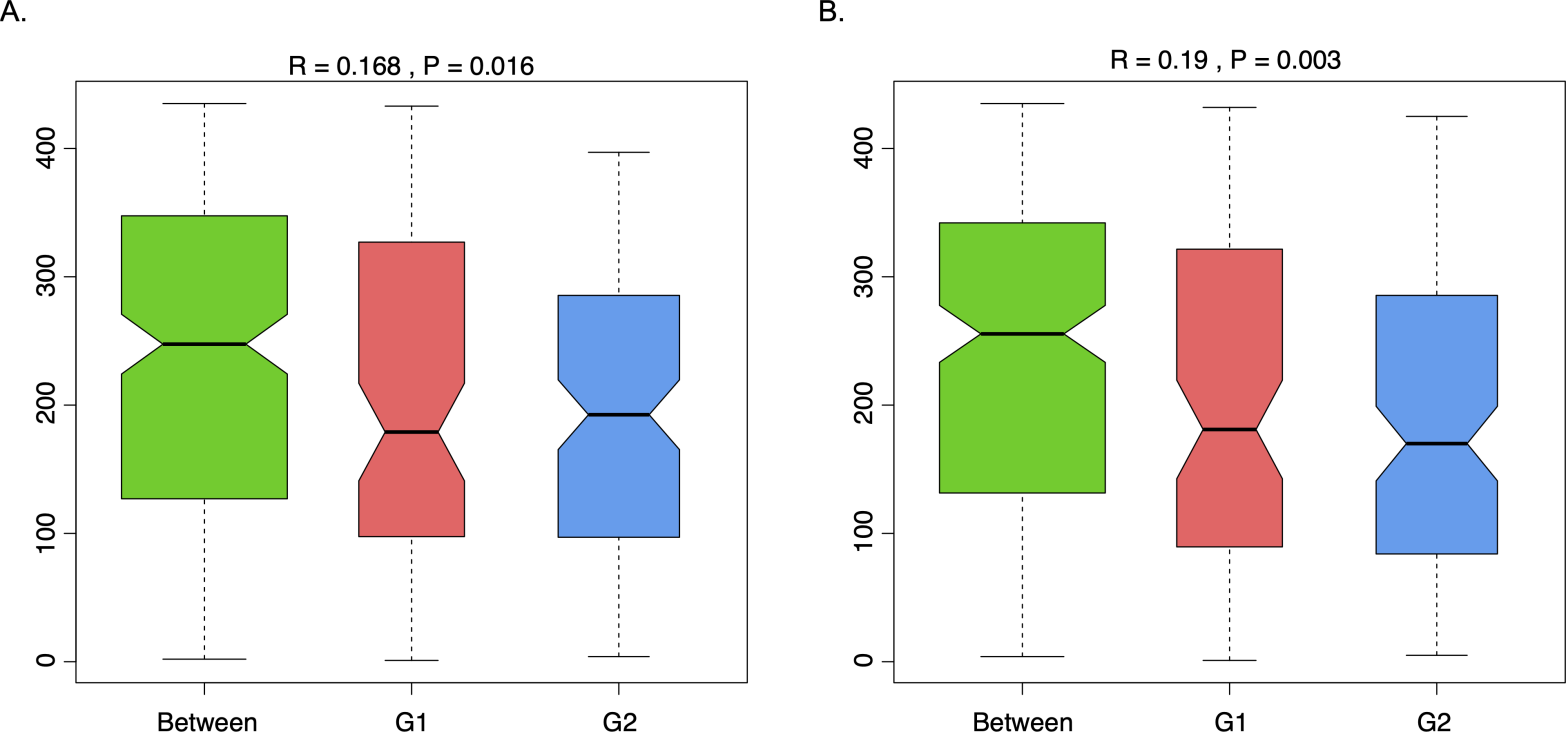
Dissimilarities between the low (G1) and high (G2) *S. cristatus-P. gingivalis* ratio groups. The ANOSIM test was used to compare the mean ranked dissimilarities between the two groups at the (**A**) genus and (**B**) species levels. The green box represents dissimilarities of sample pairs between the two groups, whilst the red and blue boxes represent those within G1 and G2, respectively. Positive *R* values of 0.168 and 0.19 and a *P*-value < 0.05 indicate that the inter-group variation is statistically more significant than the intra-group variation.

**Fig 3:**
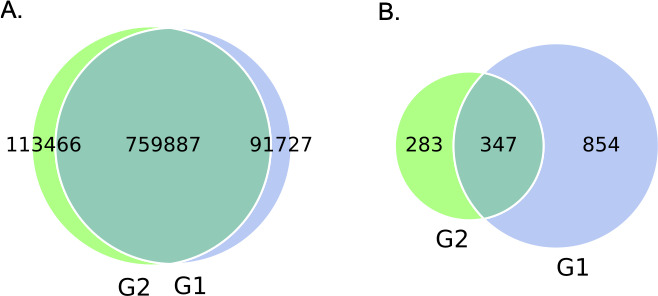
Differences in genes and taxonomy between groups. (**A**) Total genes annotated in groups with low (G1) or high (G2) *S. cristatus-P. gingivalis* ratios are presented in a Venn figure. Green and blue areas represent the number of peculiar genes identified within each group, respectively, and an overlap area represents the number of common genes found in both groups. (**B**) Taxonomic difference between the groups at the species level.

A total of 965,080 genes were identified using MetaGeneMark (20, 31). Figure 1 shows the mean (238,472) and median (216,072) of non-redundant genes in samples with low *S. cristatus-P. gingivalis* ratios. While the mean and median gene counts were 294,355 and 316,927, respectively, in samples with high *S. cristatus-P. gingivalis* ratios. Although a statistically significant difference in numbers of the non-redundant genes and total taxonomy counts (Table S1) was not reached between the two groups, significant abundance dissimilarities were observed at the genus (*R* = 0.168 and *P* = 0.016) and species levels (*R* = 0.19 and *P* = 0.003) between these groups using ANOSIM analysis, a non-parametric test based on the ranked dissimilarity measure (Fig. 2). This suggests a higher similarity of gene abundance within each group than the similarity between the groups at the genus and species levels. Moreover, some non-redundant genes were observed in both groups as shown in Venn diagrams (Fig. 3A). Among the 965,080 non-redundant genes identified, 91,727 genes (9.50%) were unique in the group with low *S. cristatus-P. gingivalis* ratios (G1), while 113,466 (11.75%) were observed only in the group with the high ratios (G2). Furthermore, taxonomic annotations identified 1,484 microbial species in the cohort. Microbial taxa were found to be more diverse in the samples with low *S. cristatus-P. gingivalis* ratios. A total of 1,201 species were identified in the group with low *S. cristatus-P. gingivalis* ratios, while only 630 species were identified in the group with high *S. cristatus-P. gingivalis* ratios. Among the 1,201 microbial taxa annotated in the group with low *S. cristatus-P. gingivalis* ratios, 854 group-specific species (58.6%) were found compared to 283 unique species (19.1%) in the group with the high ratios. A total of 347 species were annotated in both groups. These results indicate a more complex microbial profile in the group with low *S. cristatus-P. gingivalis* ratios (Fig. 3B).

**Fig 4:**
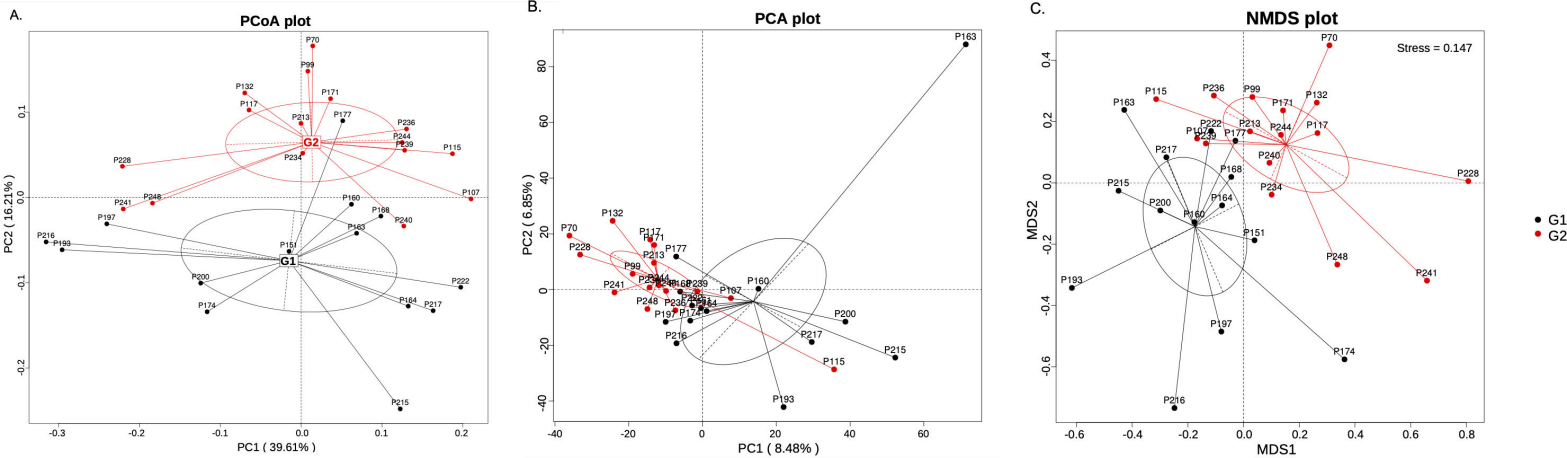
(**A**) PCoA, (**B**) PCA, and (**C**) NMDS data plots at the species level. Black dots represent the samples with a low *S. cristatus-P. gingivalis* ratio, while the red dots denote the samples high ratio. In plots (**A**) and (**C**), the distance between each pair of samples represents the dissimilarity between them.

To identify potential patterns or correlations between two groups of samples, we also conducted PCoA, PCA, and NMDS analyses. The PCA analysis transformed the high-dimensional data of samples into a lower-dimensional space while retaining the original variability. It is effective when the relationships between samples are linear. Other two-dimensionality-reduction methods, PCoA and NMDS, however, do not assume linear relationships. The visualizations of their results are provided in Fig. 4. The distance between each sample pair indicates their dissimilarity. These results showed a clear distinction between the two groups with different *S. cristatus-P. gingivalis* ratios (G1 and G2), with the samples within each group clustered together (Fig. 4A and C). The PCA plot shows similar overall separation of the two groups (Fig. 4B).

### Abundance of common bacterial taxa

**Fig 5:**
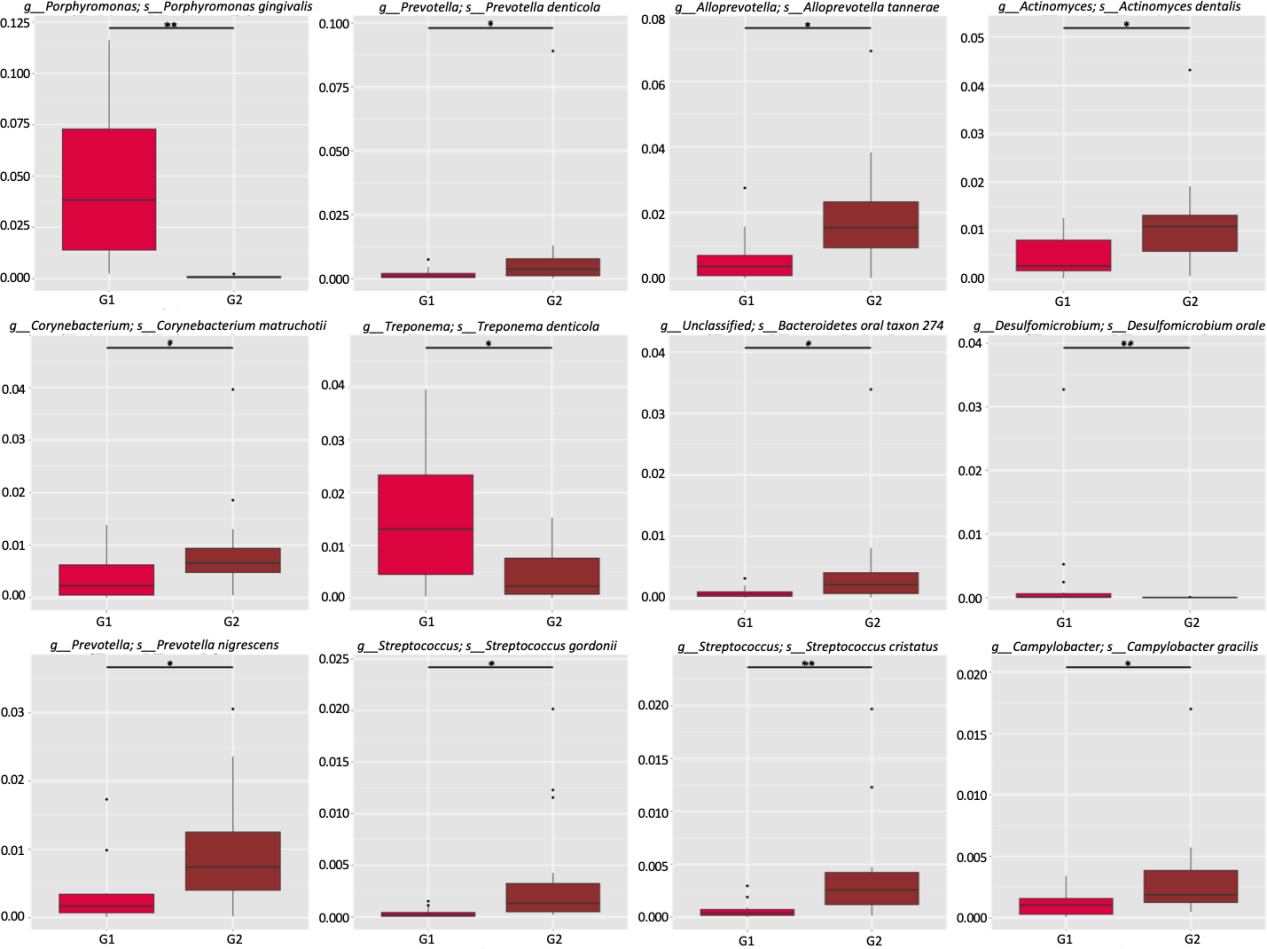
Relative abundance of the top 12 bacterial species. Oral microbial richness and evenness were determined using Metastats analysis, and the differences between the low (G1) or high (G2) *S. cristatus-P. gingivalis* ratio groups were determined using a non-parametric *t*-test. *P < 0.05 and **P < 0.01.

**Fig 6:**
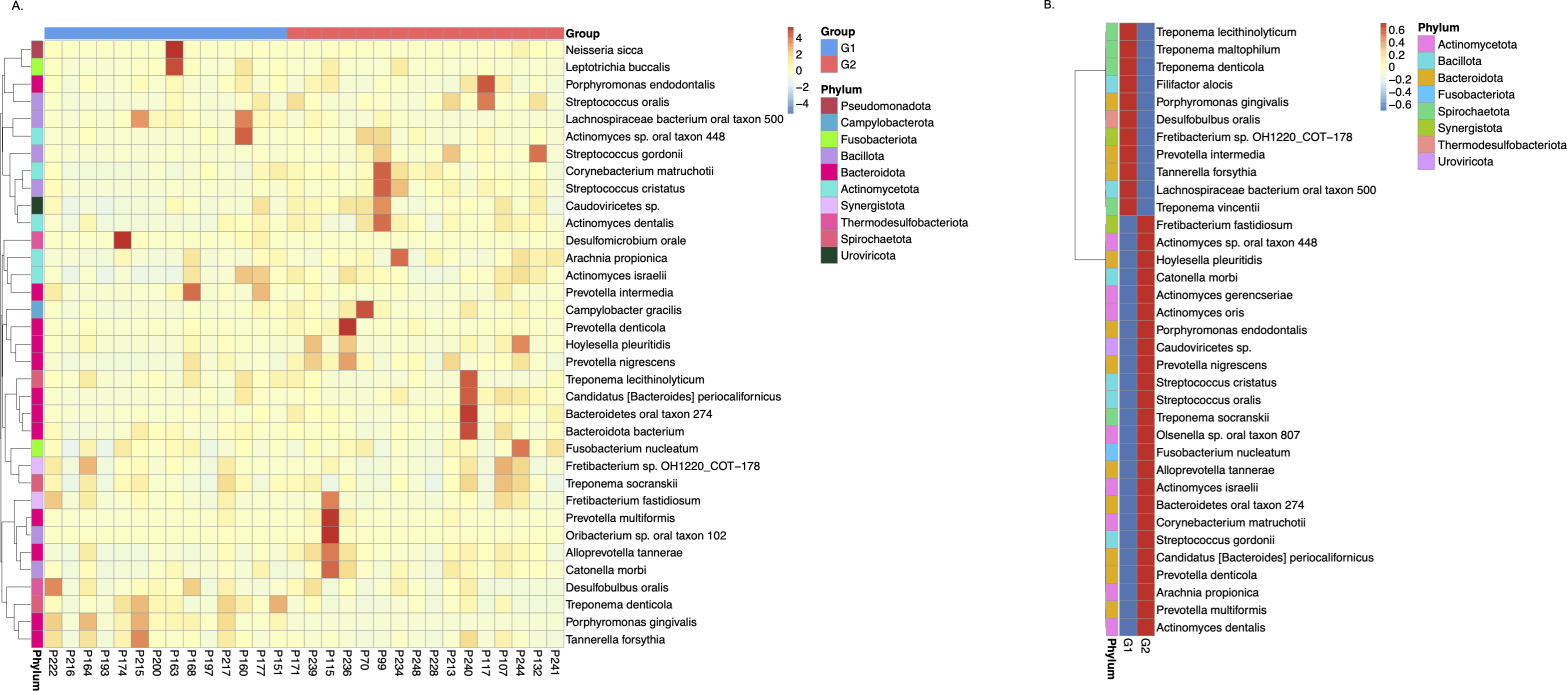
Heatmap showing the abundance of the top 35 variant species derived from the metagenomic sequencing data. (**A**) Heatmap of all 30 samples. *X*-axis represents the sample information, *Y*-axis represents species annotation, and the left side of heatmap is the species cluster tree. The value of the heatmap is a standardized *Z* score. (**B**) Heatmap by groups. Columns represent samples in the two groups. G1 is the group with low *S. cristatus-P. gingivalis* ratios, and G2 is with high ratios. The red color represents a higher value of each species’ abundance compared to that with a blue color.

Metastats (32) was performed to detect the differential abundance of common oral bacteria between the two groups with different *S. cristatus-P. gingivalis* ratios. As shown in Fig. 5, three bacterial species, *P. gingivalis*, *Treponema denticola*, and *Desulfomicrobium orale*, out of the 12 most abundant taxa, were detected 56.08-, 5.83-, and 5.62-fold more abundant, respectively, in samples with low *S. cristatus-P. gingivalis* ratios, compared to the samples with high ratios. The other nine common bacterial taxa (*Prevotella denticola*, *Alloprevotella tannerae*, *Actinomyces dentalis*, *Corynebacterium matruchotii*, *Bacteroidetes* oral taxon 274, *Prevotella nigrescens*, *Streptococcus gordonii*, *S. cristatus*, and *Campylobacter gracilis*) were 1.80- to 6.77-fold more abundant in samples with the high ratios than those with the low ratios. In addition, the top 35 microbial taxa with differential abundances between groups are shown in an abundance heatmap (Fig. 6A). Among them, 11 species exhibit a higher abundance in the group with lower *S. cristatus-P. gingivalis* ratio, compared to the group with a high ratio (Fig. 6A and B). These observations are consistent with previous studies of microbial profiles of dental plaques (33, 34), where a relatively higher abundance of *P. gingivalis* was found with elevated levels of *T. denticola*, *Tannerella forsythia*, *Filifacter alocis* in the group with low *S. cristatus-P. gingivalis* ratios, compared to those found in the group with high ratios. Other bacterial species found in more abundance in the group with low ratios were *Treponema lecithinolyticum*, *Treponema maltophilum*, *Treponema vincentii*, *Desulfobulbus oralis*, *Fretibacterium* sp. OH1220_COT-178, *Prevotella intermedia*, and *Lachnospiraceae bacterium* oral taxon 500 (Fig. 6B). In contrast, *Streptococcus* spp. and *Actinomyces* spp. were dominant in the samples with the higher ratios, in which more than sixfold higher level of *S. cristatus* was discovered. Notably, hierarchical clustering algorithms showed that along with *T. denticola* and *T. forsythia*, another bacterial species, *D. oralis* was often positively co-occurrent with *P. gingivalis* in the samples (Fig. 6A). While *S. cristatus* was clustered with *C. matruchotii*, *Actinomyces dentalis,* and *S. gordonii*. Interestingly, dsDNA bacteriophages of *Caudoviricetes* sp., which are highly prevalent in the human gastrointestinal tract (35), clustered with *S. cristatus*. Overall, these results revealed differential levels of microbial taxa in the samples with low or high *S. cristatus-P. gingivalis* ratios, respectively, suggesting that different core microbiota are constructed based on the ratio of *S. cristatus-P. gingivalis*.

### Functional profile of oral microbiome with different *S. cristatus-P. gingivalis* ratios

**Fig 7:**
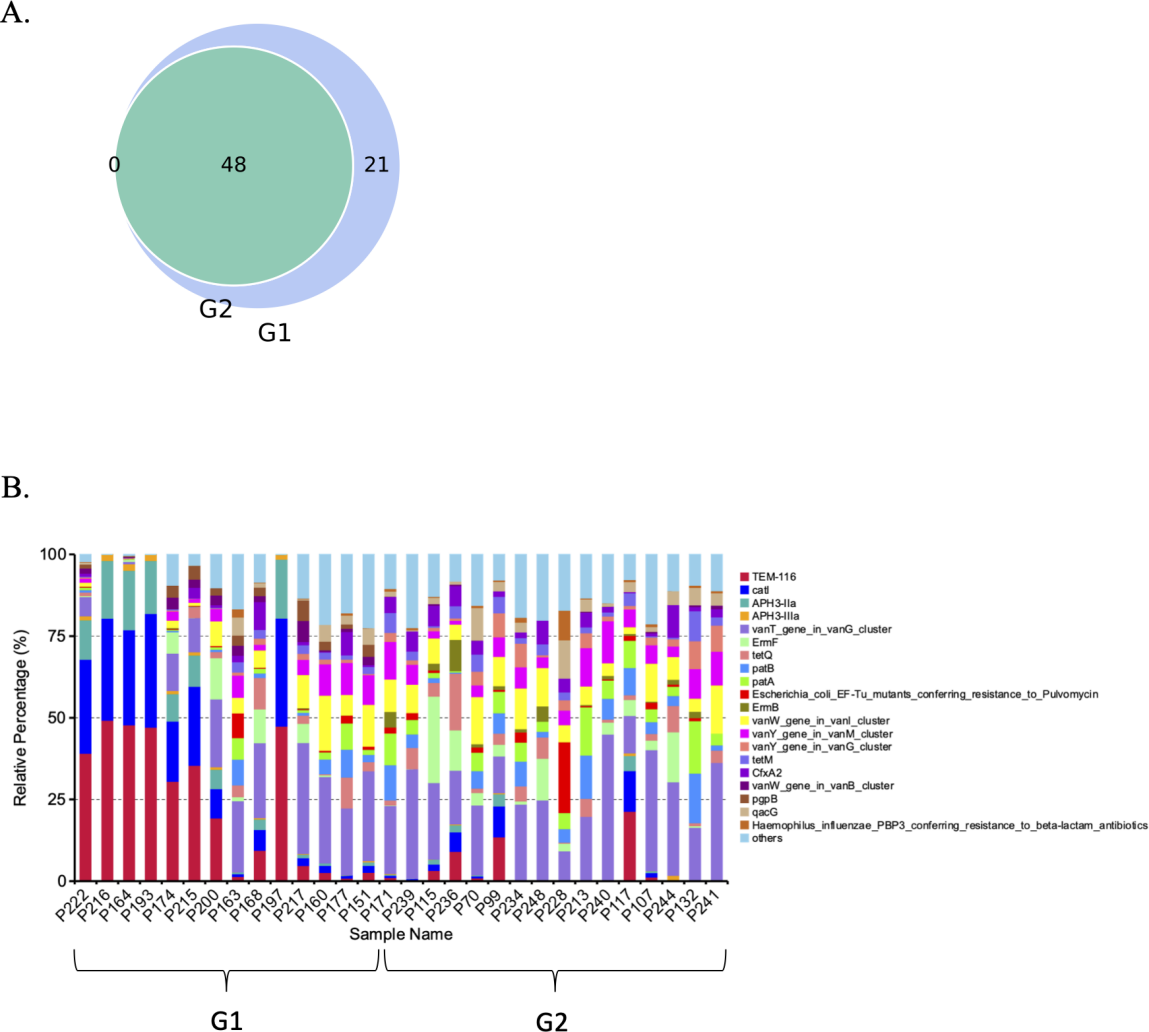
Identification of antibiotic resistance genes in the dental plaque samples. (**A**) Total antibiotic-resistant genes identified in the 30 samples. The blue circle represents the number of antibiotic-resistant genes detected in the low ratio group, while the green circle shows those detected in the high ratio group. The overlapping area shows the common genes. (**B**) Each stacked bar represents the relative abundance of antibiotic resistance genes in each sample.

To determine the functional diversity of bacterial communities in the two groups with low and high *S. cristatus-P. gingivalis* ratios, we mapped functional annotation against several functional databases including CARD (25), CAZy (26), KEGG (27), and eggNOG (28). As shown in Fig. 7A, 69 antibiotic resistance genes were identified in the 30 dental plaque samples, among which 48 were found in all samples with different abundance levels. Although with relatively low abundance, 21 antibiotic resistance genes, including multidrug efflux pump membrane fusion proteins (mdtA, B, F, and G), were unique to the group with low *S. cristatus-P. gingivalis* ratios. The percentage of antibiotic resistance genes in each sample is indicated in Fig. 7B, and absolute copies of antibiotic resistance genes are listed in Table 2. Among the top 30 antibiotic resistance genes annotated, six were significantly abundant in samples with lower *S. cristatus-P. gingivalis* ratios compared to those with higher ratios, while seven genes were more prevalent in the samples with higher ratios. The antibiotic resistance genes that exhibit remarkably differential abundance between the two groups include *Tem*-116, *catI*, *aph3-IIa*, and *aph3-IIIa* with increased levels in the samples with the low *S. cristatus-P. gingivalis* ratios (Table 2). These results of the differential richness of antibiotic resistance genes in the two groups indicate a potential regulatory mechanism contributing to the physiology of the oral microbiome.

**TABLE 2:** Abundance of antibiotic resistance genes in the low and high *S. cristatus-P. gingivalis* ratio groups

Antibiotic resistance genes	Gene function	Total gene copies		Fold changes	*P*-value
		G1	G2	G1/G2	
*tem-116*	β-lactamase β-lactam antibiotics	24,527.77	42.29	580.03	0.045
*catI*	Chloramphenicol acetyltransferase Chloramphenicol	16,825.01	26.61	632.28	0.045
*aph*3-IIa	Aminoglycoside phosphotransferase	8,841.63	10.39	851.09	0.045
*aph*3-IIIa	Aminoglycoside-3′- phosphotransferase Kanamycin	860.43	2.22	388.38	0.045
*van*T_gene_in_*van*G _cluster	Resistance to vancomycin and teicoplanin-type antibiotics	114.94	149.04	0.77	0.164
*erm*F	Member of RNA methyltransferase family	26.46	45.72	0.58	0.349
*tet*Q	Tetracycline-resistant ribosomal protection protein	25.05	33.31	0.75	0.586
*pat*B	Associated with fluoroquinolone resistance	14.38	44.64	0.32	0.023
*pat*A		12.42	45.82	0.27	0.014
*Escherichia coli*_EF-Tu_mutants_conferring_ resistance_to_Pulvomycin	Resistance to pulvomycin	7.54	14.66	0.51	0.399
ErmB	A member of RNA methyltransferase family	2.86	15.25	0.19	0.123
*van*W_gene_in_*van*I_cluster	Resistance to vancomycin and teicoplanin-type antibiotics	31.52	50.32	0.63	0.031
*van*Y_gene_in_*van*M_cluster		25.54	42.52	0.60	0.073
*van*Y_gene_in_*van*G_cluster		5.58	23.15	0.24	0.010
*tet*M	Tetracycline-resistant ribosomal protection protein	8.40	24.48	0.34	0.026
*cfx*A2	Beta-lactamase	17.17	26.61	0.65	0.271
*van*W_gene_in_*van*B_cluster	Resistance to vancomycin and teicoplanin-type antibiotics	21.36	1.59	13.45	0.001
*pgp*B	Phosphatidylglycerophosphatase	22.91	0.05	441.68	0.000
*qac*G	Resistance to benzalkonium chloride and ethidium bromide	9.82	23.83	0.41	0.039
*Haemophilus_influenzae* _PBP3_conferring_resistance _to_beta-lactam_antibiotics	Resistance to beta-lactam antibiotics	2.92	6.37	0.46	0.320
nimI	Nitroimidazole reductase	6.83	10.44	0.65	0.425
*van*W_gene_in_*van*G_cluster	Resistance to vancomycin and teicoplanin-type antibiotics	7.36	6.04	1.22	0.705
*tet*W	A protein binds to the 30S ribosomal subunit	1.03	5.68	0.18	0.061
*van*Y_gene_in_*van*B_cluster	Resistance to vancomycin and teicoplanin-type antibiotics	7.78	11.10	0.70	0.270
*mde*A	Multidrug efflux pump	2.02	7.44	0.27	0.077
*tet*32	Tetracycline-resistant ribosomal protection protein	5.87	6.40	0.92	0.807
*van*Y_gene_in_*van*F_cluster	Resistance to vancomycin and teicoplanin-type antibiotics	12.66	10.04	1.26	0.403
*Klebsiella pneumoniae*_*kpn*H	Pumping of antibiotic out of a cell to confer resistance	0.76	3.29	0.23	0.104
*nim*J	Deactivation of nitroimidazole antibiotics	1.51	6.16	0.25	0.007
*ade*F	Antibiotic efflux	4.03	3.92	1.03	0.941

**Fig 8:**
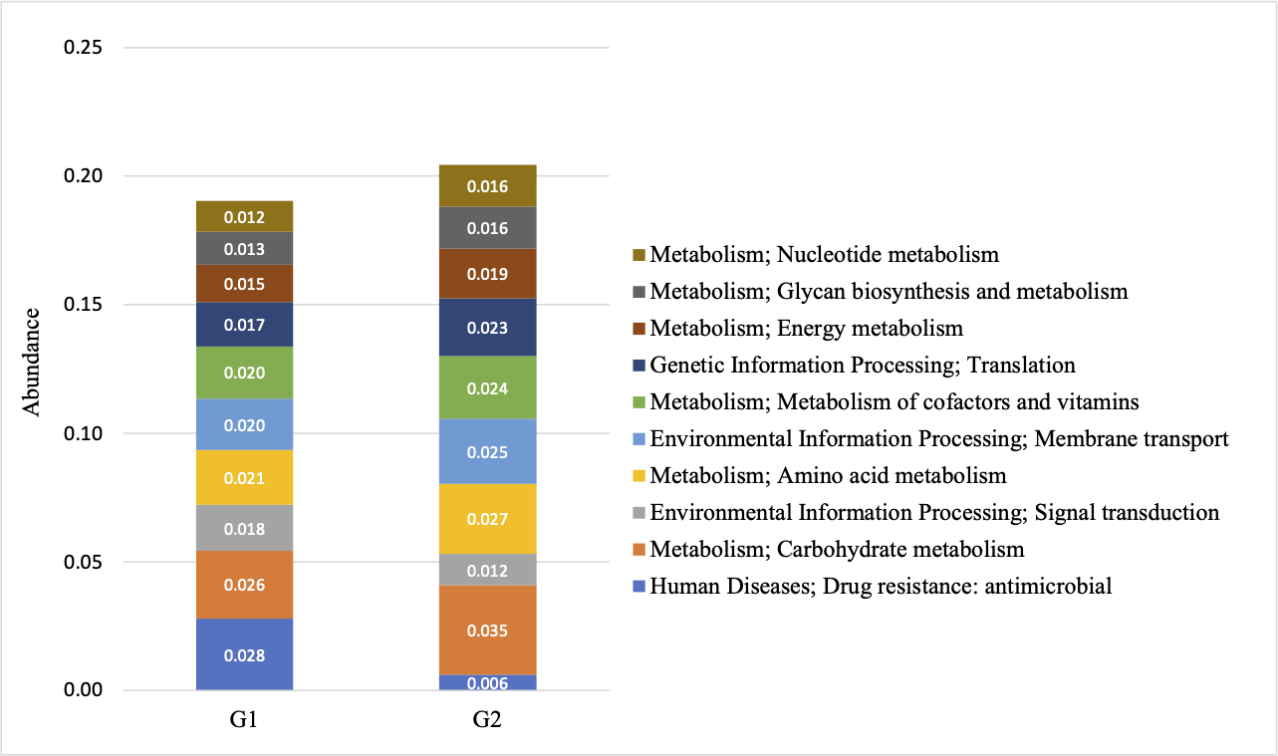
The functional diversities of groups with low (G1) or high (G2) *S. cristatus-P. gingivalis* ratios. Metagenomic proteins were quantified by annotating metagenomic sequences with functions. Protein coding sequences were mapped against functional databases. Stacked bar represents the proportion of relative functional abundance.

**Fig 9:**
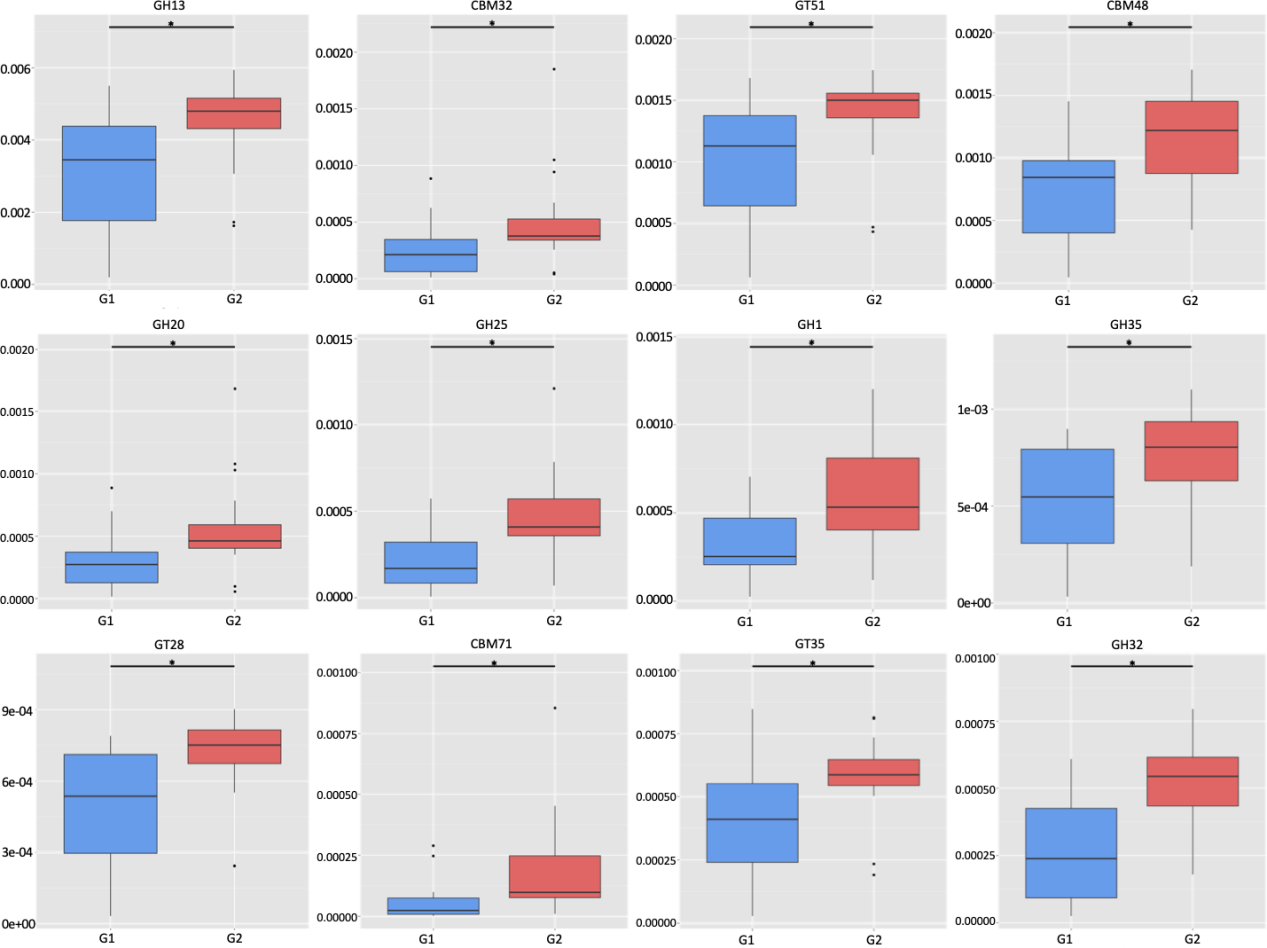
Abundance of carbohydrate-active enzymes in the two groups. Box plots represent the richness and evenness of the top 12 genes encoding enzymes involved in bacterial metabolism. An asterisk refers to the significant difference between the two groups. GH, glycoside hydrolase family; CBM, carbohydrate-binding module family; and GT, glycosyl transferase family.

In light of the observations on the differential abundance of functional genes, we then investigated the molecular interaction and reaction networks using KEGG pathway databases and compared them between the samples with low or high ratios of *S. cristatus-P. gingivalis*. The results revealed that the genes involved in human disease-associated antimicrobial drug resistance were enriched in the samples with the low ratios (*P* = 0.0498) (Fig. 8). The genes encoding enzymes essential for carbohydrate metabolism were more abundant in the samples with high ratios (*P* = 0.0324), which is in agreement with the results of analyses using the CAZy database (Fig. 9). Furthermore, the functional profile of the oral microbiome showed significant variations in abundance of genes involved in carbohydrate processing between the groups with different *S. cristatus-P. gingivalis* ratios, including a reduction in some families of glycoside hydrolases, carbohydrate-binding modules, and glycosyl transferases in the samples with low ratios of *S. cristatus-P. gingivalis*. Moreover, the genes involved in the metabolic pathways of amino acids (*P* = 0.040), energy (*P* = 0.0463), amino acid metabolism (*P* = 0.0403), and nucleotide (*P* = 0.0296), as well as glycan biosynthesis and metabolism (*P* = 0.0495), were significantly abundant in the group with high ratios (Fig. 8). However, there is no significant difference in the pathways of environmental information processing (signal transduction and membrane transport), genetic information processing (translation), metabolism of cofactors and vitamins. These observations suggest a potential association of *S. cristatus-P. gingivalis* ratios with functional variation in the oral microbiome.

## DISCUSSION

A series of studies in our laboratory have established a specific role of *S. cristatus* in the virulence potential of *P. gingivalis* and in the formation and composition of oral microbial biofilms using qPCR (9, 10, 14, 15, 30, 36). Although we previously reported that *S. cristatus* and *P. gingivalis* were negatively correlated in dental plaque samples derived from patients with periodontitis (15), it is currently not clear how this relationship influences microbial communities as a whole. The study presented here provides a first glance at intraspecies interactions associated with different ratios of *S. cristatus-P. gingivalis* using metagenomic shotgun sequencing. Besides the identification of over 1,400 microbial taxa at the species level in plaque samples from 30 periodontitis patients, this approach revealed significant differences in abundance, diversity, and functions between two groups of dental plaque samples with high or low *S. cristatus-P. gingivalis* ratios, respectively. Earlier studies by Dewhirst et al. (37, 38) estimated, using 16S rRNA sequencing, that there may be about 600 common taxa in the human oral microbiome. With powerful shotgun metagenomic sequencing here, we successfully identified over 1,400 microbial species, including ones at a very low abundance, therefore, enabling future investigation of potential roles of low prevalent taxa in oral health and diseases.

Our study also revealed significant differences in the abundance of common bacterial species between two groups with different *S. cristatus-P. gingivalis* ratios. Several well-known periodontitis-associated bacteria, including *T. denticola*, *T. forsythia*, and *F. alocis*, were much abundant in the samples with low *S. cristatus* and *P. gingivalis* ratios. Along with low *S. cristatus* and *P. gingivalis* ratios, we found a close correlation of abundance among *P. gingivalis*, *T. forsythia*, *T. denticola*, and *D. oralis* in the samples tested. The correlation is likely due to the coaggregation between/among these bacterial species. The molecular mechanisms of coaggregation between *P. gingivalis* and *T. denticola* have been found to include the interaction of *P. gingivalis* fimbrial protein and *T. denticola* dentilisin (39). Moreover, *T. denticola* dentilisin is also known to be responsible for the coaggregation of *T. denticola* and *T. forsythia* (40). *P. gingivalis* Hgp44 may also be involved in the adhesion of *P. gingivalis* to *T. denticola*, as a truncated Hgp446 fragment reduced the coaggregation of *P. gingivalis* and *T. denticola* (41). A similar observation reported by Zhu et al. indicated an essential role of *P. gingivalis* gingipains in synergistic polymicrobial biofilm formation of *P. gingivalis* and *T. denticola* (42). Unlike the recognized roles of *P. gingivalis*, *T. denticola*, and *T. forsythia*, the role of *D. oralis* in the pathogenesis of periodontitis is unclear. *D. oralis*, a Gram-negative, non-motile rod-shaped bacterium, was first isolated and purified from subgingival plaque samples from a periodontitis patient (43). The pathogenicity of *D. oralis* may rely on its ability to stimulate the production of IL-1β, IFN-α, IFN-γ, MCP-1, IL-6, IL-8, and IL-1 by oral keratinocyte cells (43), suggesting that the organism plays a role in periodontal inflammation. Although there is no evidence that *D. oralis* physically interacts with *P. gingivalis*, *T. forsythia*, and/or *T. denticola*, our discovery of a cluster abundance of these four bacteria and increased levels of all four species in the samples with low ratios of *S. cristatus-P. gingivalis* indicates that *D. oralis* may cooperate with well-known periodontitis-associated bacteria as a core microbiota responsible for etiology and progression of periodontitis.

The shotgun metagenomic sequencing also allowed us to identify 69 antibiotic resistance genes in the dental plaques from the 30 patients with periodontitis. Among these genes, 48 were found in both tested groups and an additional 21 were only found in the group with low *S. cristatus-P. gingivalis* ratios. Additionally, four out of five most abundant antibiotic resistance genes, *tem-116*, *cat*I, *aph*3-IIa, and IIIa, were predominantly detected in the group with low *S. cristatus-P. gingivalis* ratios compared to their counterparts. The observation of abundant genes in the group with low ratios may result from the diversity of microorganisms in the samples in this group. Another explanation is that the low ratio of *S. cristatus-P. gingivalis*-associated microbiome creates an environment facilitating the transmission of resistance genes. Horizontal gene transfer is known as a major pathway for spreading antibiotic resistance genes, e.g., an outbreak of multidrug-resistant nosocomial pathogens caused by a transferable plasmid encoding SHV-12 extended-spectrum β-lactamase (TEM-116) (44). Our discovery of more diverse and abundant antibiotic resistance genes in the low ratio group implies the important role of *S. cristatus-P. gingivalis* ratios in controlling the pathogenicity of oral microbial communities. It is likely that decreased *S. cristatus-P. gingivalis* ratios facilitate the selection of antibiotic-resistant strains in the oral microbiome, which elevates the susceptibility of individuals with relatively low ratios to periodontitis. The ratios of *S. cristatus-P. gingivalis* levels also appear to shape the metabolic function of the oral microbiome. All top 12 carbohydrate metabolic pathways were found to be relatively abundant in the samples with higher *S. cristatus-P. gingivalis* ratios. Although a direct association between metabolic activity and the virulence potential of the oral microbiome has not been established, it is speculated that a decreased metabolism of carbohydrates inhibits the growth and physiological activities of the beneficial microbes.

As reported in several studies of 16S rRNA sequencing (45, 46), the abundance and diversity of several bacterial genera shifted during periodontal health and disease transition. The dental plaque samples in this study were collected from patients with stage II and III periodontitis. Despite no significant difference in gender, age, teeth numbers, BOP scores, and PI between the two tested groups that were designated based on their *S. cristatus-P. gingivalis* ratios, significant differences in abundance and diversity of bacterial species as well as functional pathways between the two groups exist. We postulate that *S. cristatus-P. gingivalis* ratios associated with an increase in abundance and diversity of periodontitis-associated bacteria and antibiotic resistance genes accelerate the progression of periodontitis and reduce response to periodontitis treatment. This notion is supported by our previous observations that the ratio of *S. cristatus-P. gingivalis* was significantly higher in CAs than in AAs and that better gains in clinical attachment levels were observed in CA periodontitis patients compared to those found in AAs after nonsurgical periodontal treatment (14, 29).

In conclusion, our work highlights the role of *S. cristatus-P. gingivalis* ratios in the virulence potential of the oral microbiome. Microbial communities with low *S. cristatus-P. gingivalis* ratios had elevated levels of several well-studied periodontitis-associated bacteria, decreased levels of *Streptococcus* spp. and *Actinomyces* spp., and a diverse microbial composition and antibiotic-resistance gene profiles. Diversity and abundance of the oral microbiome are likely regulated by the core bacteria, which include *S. cristatus* and *P. gingivalis*, and may lead to functional changes in the oral microbiome. Therefore, approaches for modulating the *S. cristatus-P. gingivalis* ratio may be significant in maintaining a healthy oral microbiome.

## Data Availability

Our metagenomic sequencing data are deposited at Sequence Read Archive (SRA) with accession number PRJNA1010809.

## SUPPLEMENTAL MATERIAL

Table S1 spectrum.03482-23-s0001.docx The counts of taxonomies identified in plaque samples and abundance of two bacteria as measured by the total number of reads mapped to bacterial genomes.

ASM does not own the copyrights to Supplemental Material that may be linked to, or accessed through, an article. The authors have granted ASM a non-exclusive, world-wide license to publish the Supplemental Material files. Please contact the corresponding author directly for reuse.
