# Supplementary material for: Oral microbiome associated with differential ratios of Porphyromonas gingivalis and Streptococcus cristatus
Source: Microbiol Spectr. 2024 Jan 17;12(2):e03482-23. doi: 10.1128/spectrum.03482-23 (PMC10846039; doi:10.1128/spectrum.03482-23)
Supplement: Table S1 — The counts of taxonomies identified in plaque samples and abundance of two bacteria as measured by the total number of reads mapped to bacterial genomes. [file spectrum.03482-23-s0001.docx]

**Supplement Table 1. The counts of taxonomies identified in plaque samples and abundance of two bacteria as measured by the total number of reads mapped to bacterial genomes.**

| **G1** | | | | | **G2** | | | | |
| --- | --- | --- | --- | --- | --- | --- | --- | --- | --- |
| **Samples** | **Taxonomy count** | ***S. cristatus* (S) abundance** | ***P. gingivalis* (P) abundance** | **S/P** | **Samples** | **Taxonomy count** | ***S. cristatus* (S) abundance** | ***P. gingivalis* (P) abundance** | **S/P** |
| P222 | 3,467 | 1,040 | 2,575 | 0.40 | P171 | 2,298 | 1,004 | 371 | 2.71 |
| P216 | 1,629 | 73 | 1,841 | 0.04 | P239 | 3,601 | 829 | 803 | 1.03 |
| P164 | 2,435 | 174 | 2,314 | 0.08 | P115 | 3,598 | 1,045 | 509 | 2.05 |
| P193 | 2,905 | 196 | 2,290 | 0.09 | P236 | 3,214 | 966 | 263 | 3.67 |
| P174 | 1,318 | 70 | 1,994 | 0.04 | P70 | 2,540 | 1,373 | 840 | 1.63 |
| P215 | 2,851 | 210 | 2,370 | 0.09 | P99 | 2,737 | 1,190 | 293 | 4.06 |
| P200 | 2,410 | 128 | 2,243 | 0.06 | P234 | 2,333 | 1,059 | 230 | 4.60 |
| P163 | 4,434 | 953 | 2,511 | 0.38 | P248 | 1,543 | 379 | 204 | 1.86 |
| P168 | 2,568 | 239 | 2,228 | 0.11 | P228 | 1,373 | 674 | 125 | 5.39 |
| P197 | 1,792 | 576 | 2,138 | 0.27 | P213 | 2,916 | 1,126 | 678 | 1.66 |
| P217 | 2,986 | 558 | 2,379 | 0.23 | P240 | 2,493 | 565 | 446 | 1.27 |
| P160 | 2,416 | 221 | 1,734 | 0.13 | P117 | 2,131 | 1,105 | 234 | 4.72 |
| P177 | 2,893 | 847 | 2,178 | 0.39 | P107 | 3,095 | 937 | 764 | 1.23 |
| P151 | 2,136 | 233 | 2,087 | 0.11 | P244 | 2,413 | 821 | 292 | 2.81 |
|  |  |  |  |  | P132 | 2,426 | 1,152 | 338 | 3.41 |
|  |  |  |  |  | P241 | 1,205 | 74 | 153 | 0.48 |
| **Mean** | 2,589 | 394 | 2,206 | 0.17 |  | 2,495 | 894 | 409 | 2.66 |
| **Median** | 2,502 | 227 | 2,236 | 0.11 |  | 2,460 | 985 | 316 | 2.38 |
